# Ionizing Radiation in Glioblastoma Initiating Cells

**DOI:** 10.3389/fonc.2013.00074

**Published:** 2013-04-08

**Authors:** Maricruz Rivera, Kumar Sukhdeo, Jennifer Yu

**Affiliations:** ^1^Department of Stem Cell Biology and Regenerative Medicine, Lerner Research Institute, Cleveland ClinicCleveland, OH, USA; ^2^Department of Molecular Medicine, Lerner College of Medicine of Case Western Reserve UniversityCleveland Clinic, Cleveland, OH, USA; ^3^Department of Pathology, School of Medicine, Case Western Reserve UniversityCleveland, OH, USA; ^4^Department of Radiation Oncology, Cleveland ClinicCleveland, OH, USA

**Keywords:** glioma initiating cells, radiation, c-MET, NOTCH, DNA damage response

## Abstract

Glioblastoma (GBM) is the most common primary malignant brain tumor in adults with a median survival of 12–15 months with treatment consisting of surgical resection followed by ionizing radiation (IR) and chemotherapy. Even aggressive treatment is often palliative due to near universal recurrence. Therapeutic resistance has been linked to a subpopulation of GBM cells with stem cell-like properties termed GBM initiating cells (GICs). Recent efforts have focused on elucidating resistance mechanisms activated in GICs in response to IR. Among these, GICs preferentially activate the DNA damage response (DDR) to result in a faster rate of double-strand break (DSB) repair induced by IR as compared to the bulk tumor cells. IR also activates NOTCH and the hepatic growth factor (HGF) receptor, c-MET, signaling cascades that play critical roles in promoting proliferation, invasion, and resistance to apoptosis. These pathways are preferentially activated in GICs and represent targets for pharmacologic intervention. While IR provides the benefit of improved survival, it paradoxically promotes selection of more malignant cellular phenotypes of GBM. As reviewed here, finding effective combinations of radiation and molecular inhibitors to target GICs and non-GICs is essential for the development of more effective therapies.

## Introduction

**Figure 1:**
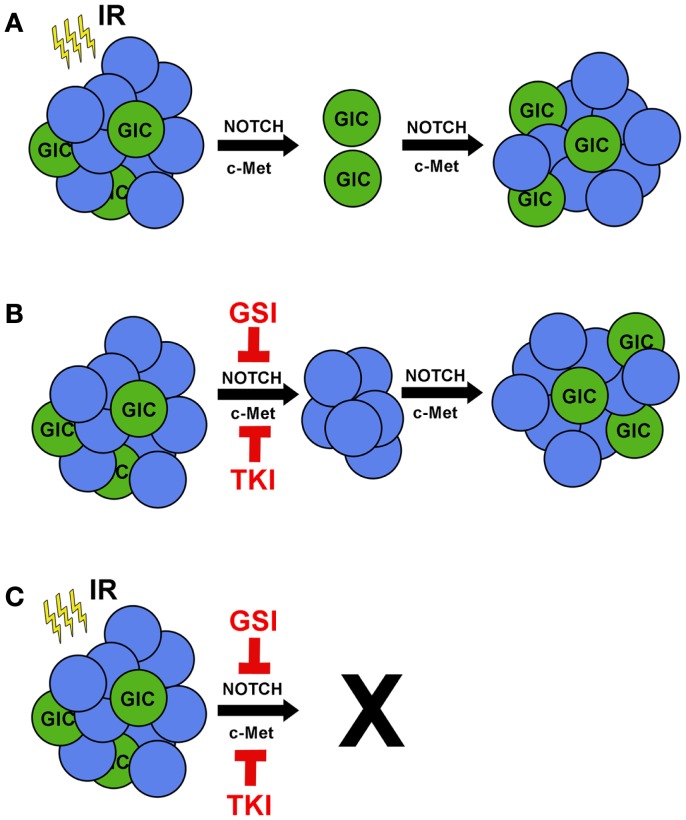
**Ionizing radiation in combination with c-MET or NOTCH inhibitors prevents tumor recurrence**. **(A)** Treating GBM with IR reduces tumor volume, but radioresistant GICs remain. IR promotes activation of the pro-survival pathways NOTCH and c-MET in GICs, leading to tumor recurrence. **(B)** Single treatment of GBM tumors with either gamma secretase inhibitors (GSIs) to target NOTCH or tyrosine kinase inhibitors (TKIs) to target c-MET would kill GICs specifically and have a minor effect on tumor volume. **(C)** Combinatorial treatment of GSIs or TKIs with IR would target both GICs and non-GICs and prevent tumor recurrence.

Glioblastoma (GBM) is the most common and aggressive type of primary brain cancer in adults with approximately 18,000 patients diagnosed each year [(http://www.CBTRUS.org); Schwartzbaum et al., 2006]. GBM can arise as *de novo* (primary) cancer or may progress from lower grade gliomas (secondary). Despite aggressive multimodality treatment consisting of maximal safe resection, adjuvant chemoradiation with temozolomide, and maintenance temozolomide, median survival remains dismal at 12–15 months (Stupp et al., 2009). Patients typically respond initially to therapy, but ultimately relapse within the high-dose irradiation field (Hochberg and Pruitt, 1980; Lee et al., 1999), suggesting the presence of a subpopulation of resistant cells. While *inter*tumoral heterogeneity between patients can, in part, explain differential patient responses (Maher et al., 2006; Phillips et al., 2006; Dang et al., 2009; Yan et al., 2009; Snuderl et al., 2011), *intra*tumoral heterogeneity is now recognized as a critical factor in determining therapeutic response (Bao et al., 2006; Liu et al., 2006). GBM initiating cells (GICs) are a subgroup of cancer cells that exhibit the ability to self-renew and express putative stem cell markers such as CD133, SSEA-1 (CD15), L1CAM, and CD44^high^ (Galli et al., 2004; Singh et al., 2004; Bao et al., 2008; Son et al., 2009; Anido et al., 2010). GICs are defined functionally by their ability to repopulate the tumor upon serial transplantation (Ignatova et al., 2002; Singh et al., 2003, 2004; Galli et al., 2004). When non-GICs are assayed in parallel, these cells fail to form tumors, even when their numbers are increased by orders of magnitude. Therefore, tumor recurrence is likely due to tumorigenic GICs equipped with resistance mechanisms to survive and proliferate following therapy (Figure 1A).

The factors that influence stem-like characteristics are more complex than previously recognized. Recently, studies have revealed the microenvironmental effects of hypoxia, low glucose, low pH, and perivascular niches in promoting GIC survival, maintenance, and cellular plasticity (Gatenby and Gillies, 2004; Calabrese et al., 2007; Heddleston et al., 2009; Soeda et al., 2009; Anido et al., 2010; Charles and Holland, 2010; Seidel et al., 2010; Zhu et al., 2011). For example, hypoxia has been shown to drive expression of stem cell genes and increase the tumorigenic capacity of GICs, particularly through hypoxia inducible factors (Heddleston et al., 2009; Soeda et al., 2009; Seidel et al., 2010). These effects were also seen in acidic conditions regardless of oxygen concentration (Hjelmeland et al., 2011). Under these conditions, non-GBM initiating cells (non-GICs) can assume stem-like features and initiate tumor formation *in vivo* (Heddleston et al., 2009; Hjelmeland et al., 2011), underscoring the plasticity of GBM cells (Figure 1B). Notably, many of these pro-GIC signaling components, such as c-MET and NOTCH, are activated by radiotherapy (Wang et al., 2010; Joo et al., 2012).

Exposure to ionizing radiation (IR) elicits a preferential activation of the DNA damage response (DDR) pathway, along with enhanced DNA repair kinetics in GICs compared to their non-GIC counterparts (Bao et al., 2006). These data suggest that GICs are better able to activate the DDR in response to genotoxic stress. Radiation causes extensive cellular damage, primarily through generation of reactive oxygen species leading to DNA double-strand breaks (DSBs). Activation of the DDR signaling cascade elicits a host of cellular responses including cell cycle regulation, DNA repair, autophagy, mitotic catastrophe, necrosis, senescence, and apoptosis. Moreover, irradiated (Bao et al., 2006) and temozolomide-treated (Firat et al., 2011) GICs have a lower percentage of apoptotic cells than their non-GIC counterparts, highlighting their intrinsic therapeutic resistance (Figure 1A). This expansion of GICs has been confirmed by histological analysis of recurrent GBM after initial treatment with chemoradiation at the time of salvage surgery (Tamura et al., 2010). Many, although not all, clinical trials have failed to show a benefit to radiation dose-escalation (Chan et al., 2002), radiosurgery boost (Souhami et al., 2004), or brachytherapy boost (Laperriere et al., 1998; Selker et al., 2002). Taken together, these studies suggest that GICs can overcome even high doses of radiation (Figure 1A). While traditional therapy may initially reduce the bulk of the tumor by targeting non-GICs, it ultimately selects for the outgrowth of a more aggressive tumor through expansion of GICs. This manifests as clinical and/or radiographic progression within several months.

## Activation of the DNA Damage Response Pathway

Genotoxic stressors, including oncogenic stressors, induce DNA damage and activate the DDR pathway. The DDR pathway is a signaling cascade with multiple sensor, transducer, and effector proteins. Two such transducers are the serine/threonine protein kinases ataxia telangiectasia mutated (ATM) and ataxia telangiectasia and Rad3-related protein (ATR). ATM and ATR are members of the phosphatidylinositol 3-kinase (PI3K) family and are key regulators of DSB repair (Matsuoka et al., 2007). Upon DNA breakage, ATM senses the damage and the MRE11-RAD50-NBS1 (MRN) complex is recruited to the damaged site to accelerate phosphorylation of inactive ATM dimers. These dimers then dissociate and each phosphorylated ATM monomer further activates the protein by auto-phosphorylation in a feed-forward mechanism to activate effector proteins including CHK2 kinase (Matsuoka et al., 1998). CHK2 represents a molecular switch by directly activating various targets responsible for cell cycle progression, DNA repair, and, if the damage is extensive, apoptosis. Additionally, ATM-CHK2 activates transcription factors that alter the expression of numerous genes including the receptor tyrosine kinase *c-MET* (De Bacco et al., 2011). The implications of promoting c-MET expression will be explained below.

ATR functions in response to endogenous DNA damage; however, it may also be activated in response to DSBs induced by IR, albeit to a lesser extent than ATM. The signaling cascade activated by ATR works through a second checkpoint kinase, CHK1 (Guo et al., 2000). CHK1 and CHK2 demonstrate both overlapping and non-redundant roles, such as those affecting cell cycle progression, DNA repair, and apoptosis (Zhou and Elledge, 2000). The contributions of the ATM-CHK2 and ATR-CHK1 signaling pathways to GIC radiation resistance remain unclear. The ATM-CHK2 pathway is preferentially activated in GICs and targeting CHK1/2 results in improved response to DNA damaging agents (Bao et al., 2006). In addition, ATM overexpression in GBM patient specimens correlates with better overall survival. Taken together, these results indicate a potential role for CHK1/2 kinase inhibitors in the treatment of GBM. Indeed CHK1 inhibitors are currently being investigated in phase I trials for advanced cancers (LY2606368, Eli Lilly and Company, 2000–2013; LY2603618, Eli Lilly and Company, 2000–2013). Further studies are needed to elucidate the mechanisms by which checkpoint kinases can be therapeutic targets or have cellular-protective roles.

## c-MET

*MET* undergoes focal amplification in ∼5% of GBM patients (Maher et al., 2006; Brennan et al., 2009; Dunn et al., 2012). Overexpression of c-MET occurs in ∼29% of GBM and directly correlates with poor patient prognosis (Maher et al., 2006; Cancer Genome Atlas Research, 2008; Brennan et al., 2009; Kong et al., 2009; Verhaak et al., 2010; Snuderl et al., 2011; Dunn et al., 2012; Joo et al., 2012). c-MET becomes activated upon interaction with its ligand, hepatocyte growth factor/scatter factor (HGF/SF), which is secreted in an autocrine fashion by GICs (Joo et al., 2012). This autocrine/paracrine loop helps maintain the GIC phenotype and underscores the significance of this signaling pathway in GBM. Enrichment of c-MET^high^-expressing cells from primary GBM display stem-like characteristics including *in vivo* tumor initiation (Li et al., 2011; De Bacco et al., 2012; Joo et al., 2012). Activation of c-MET stimulates proliferation, migration, and invasion (Kong et al., 2009; Joo et al., 2012; Kim et al., 2013). c-MET also stimulates angiogenesis through the induction of vascular endothelial growth factor (VEGF) expression (Abounader et al., 1999), and resistance to bevacizumab, an anti-VEGF monoclonal antibody, occurs by c-MET activation of pro-survival and invasion mechanisms (Lu et al., 2012).

IR increases c-MET expression, activation, and ligand secretion in GBM (De Bacco et al., 2011) and GICs (Joo et al., 2012). These effects were abrogated by treatment with an ATM inhibitor (De Bacco et al., 2011). Collectively, this suggests that blocking IR-induced c-MET up-regulation may provide therapeutic benefit (Figure 1B). This hypothesis was tested both *in vitro* and in pre-clinical models by targeting c-MET receptor with genetic approaches in combination with IR. The combinatorial approach decreased cell proliferation and tumor volumes compared to IR or c-MET inhibition alone, highlighting the synergistic benefit of combined treatment (Abounader et al., 1999; Jin et al., 2011). Targeting HGF specifically with three neutralizing antibodies also decreased tumor volume (Cao et al., 2001). Furthermore, dual inhibition of c-MET receptor and HGF-ligand expression together with IR not only reduced proliferation and tumor volume, but also increased apoptosis, DNA fragmentation, and survival (Lal et al., 2005; Li et al., 2009). These findings provide a foundation for investigating c-MET inhibitors, such as cabozantinib (XL-184; Exelixis), in combination with conventional GBM therapy.

Many new drugs targeting HGF/c-MET signaling are progressing into clinical trials. Some of these studies have been completed in other solid tumors, including skin, lung, and thyroid cancers, which are often driven by similar molecular mechanisms found in GBM. Multiple c-MET pathway inhibitors are in the developmental pipeline (Liu et al., 2010). Those that have been evaluated in GBM are listed in Table 1. Most notably, cabozantinib, a pan-tyrosine kinase inhibitor with high affinity for c-MET and VEGFR2, is being tested in a phase II clinical trial for recurrent GBM with encouraging tumor responses and acceptable toxicity (Zhang et al., 2010). Other tyrosine kinase inhibitors that secondarily target c-MET are in various stages of clinical evaluation (Table 1). The HGF/c-MET pathway may also be targeted by ligand sequestration. Rilotumumab (AMG-102; Amgen), a monoclonal antibody against HGF-ligand, has shown promise in a phase II trial in patients with solid tumors (Amgen, 2012).

**Table 1:** **Clinical trials of GBM targeting c-MET or NOTCH**.

Drug	Tumor type	Target	Phase	Trial number	Outcomes	Side effects
R4733 (RO4929097), Roche	Recurrent GBM, AMO, AO	NOTCH	I/II	NCT01189240, NCT01131234, NCT01269411, NCT01122901	Terminated. Outcomes not available	Not available
Vandetanib (ZD6474), AstraZeneca	Recurrent GBM, AA, AO, AMO	RTK	I/II	NCT00441142	Ongoing. Outcomes not available	Rash, diarrhea, headache, hypertension
Cediranib (AZD2171), AstraZeneca	Recurrent GBM	RTK	II	NCT00305656	APF6 27.6%, PRR 56%, PFS 111 days, OS 226 days	Hypertension, fatigue, diarrhea
Cabozantinib (XL-184), Exelixis	Recurrent GBM	RTK	II	NCT00704288	ORR 23%, PR 23%, DoR 2.9 months	Fatigue, transaminase elevation, thromboembolic events
Dovitinib (TKI-258), Novartis	Recurrent GBM	RTK	II	NCT01753713	Ongoing. Outcomes not available	Fatigue, diarrhea, nausea
Rilotumumab (AMG-102), Amgen	Recurrent GBM	HGF	II	NCT01113398	No response	Fatigue, headache, peripheral edema

*AA, anaplastic astrocytoma; AO, anaplastic oligodendroglioma; AMO, anaplastic mixed oligoastrocytoma; GBM, glioblastoma; APF6, alive and progression-free at 6 months; PRR: partial radiographic response (>50% reduction in contrast-enhancing volume); PFS, median progression-free survival; OS, median overall survival; ORR, overall response rate; PR, partial response; DoR, median duration of response; RTK, receptor tyrosine kinase; HGF, hepatocyte growth factor*.

## NOTCH

NOTCH receptor is over-expressed in multiple types of cancer initiating cells including GICs (Rizzo et al., 2008; Wang et al., 2012). Upon DELTA/JAGGED ligand binding, the NOTCH receptor is proteolytically cleaved by γ-secretase to promote the release and subsequent nuclear translocation of the NOTCH intracellular domain (NICD) (Guruharsha et al., 2012). This event promotes activation of the PI3K/AKT pathway and expression of NOTCH-regulated genes (Stockhausen et al., 2010; Wang et al., 2010, 2012). These target genes, including *c-myc*, *hes1*, and *hey1*, are responsible for promoting self-renewal and GIC maintenance (Hitoshi et al., 2002; Jeon et al., 2008; Wang et al., 2010; Zhu et al., 2011; Guruharsha et al., 2012).

IR induction of NOTCH activation results in an expansion of GICs (Wang et al., 2010). Combining TGF-β inhibition and IR failed to induce the DDR and NOTCH activation, underlining the interplay between the DDR and NOTCH signaling pathways (Hardee et al., 2012). *In vitro* studies of glioma cells with γ-secretase inhibitors (GSIs) decreased cell proliferation, viability, and percentage of CD133-positive cells, while inducing cell death exclusively in GICs (Fan et al., 2010; Hovinga et al., 2010). Exogenous expression of NICD2 in GICs was able to rescue the phenotype even in the presence of GSIs (Wang et al., 2010). Furthermore, *in vivo* studies of GBM xenografts treated with GSIs impaired tumor growth and increased survival (Fan et al., 2010), and these effects synergized with radiation (Hovinga et al., 2010; Lin et al., 2010; Wang et al., 2010). Collectively, these data indicate that GSIs effectively target GICs and may be synergistic with IR (Figure 1C).

Currently, there are several phase I or phase I/II clinical trials examining GSIs for the treatment of patients with GBM (Table 1). RO4929097 is a GSI that has shown early promise in a phase I trial with chemoradiation for newly diagnosed glioma [Princess Margaret Hospital, National Cancer Institute (NCI), 2000b; National Cancer Institute (NCI), 2000]. Single agent or neoadjuvant use of RO4929097 has moved into a phase II trial for recurrent or progressive GBM [Sydney Kimmel Comprehensive Cancer Center, National Cancer Institute (NCI), 2000]. RO4929097 is also being used in combination with the tyrosine kinase inhibitor cediranib (AZD2171/AstraZeneca) in multiple solid tumors, including high grade gliomas [Princess Margaret Hospital, National Cancer Institute (NCI), 2000a] as well as with bevacizumab in patients with recurrent or progressive high grade gliomas NCT01189240 [National Cancer Institute (NCI), 2000]. We eagerly await the results of these studies.

## Conclusion

Glioblastoma initiating cells have evolved the ability to activate c-MET and NOTCH pathways after IR, highlighting the cunning ways by which GICs overcome standard cytotoxic treatment. Pre-clinical data on targeting of these pathways have shown potential and have led to multiple clinical trials. Ultimately, too many single agents have failed due to the presence of multiple resistance mechanisms that render single agent therapies ineffective. Combined modality therapy with radiation, chemotherapy, and inhibitors of growth factor signaling will likely be necessary to improve therapy.

## Conflict of Interest Statement

The authors declare that the research was conducted in the absence of any commercial or financial relationships that could be construed as a potential conflict of interest.
